# Tepotinib‐induced hand‐foot skin reaction

**DOI:** 10.1002/rcr2.1395

**Published:** 2024-05-28

**Authors:** Takafumi Yamano, Toshihide Yokoyama, Tadashi Ishida

**Affiliations:** ^1^ Department of Respiratory Medicine Kurashiki Central Hospital Okayama Japan; ^2^ Department of Respiratory Medicine Osaka Red Cross Hospital Osaka Japan

**Keywords:** drug side effects, hand‐foot syndrome, lung neoplasm, skin, Tepotinib

## Abstract

Tepotinib may cause hand‐foot skin reactions with keratotic changes. When such changes are observed in the hands or toes after starting tepotinib treatment, its side effects should be considered, and corticosteroid ointment or withdrawal of tepotinib should be considered if necessary.

## Graphical abstract

Herein, we report a case of tepotinib‐induced hand‐foot skin reaction (HFSR) with keratotic changes.

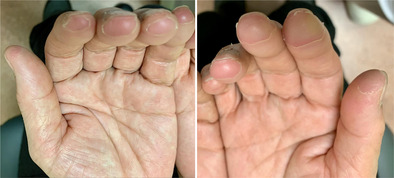

## CLINICAL IMAGE

**FIGURE 1:**
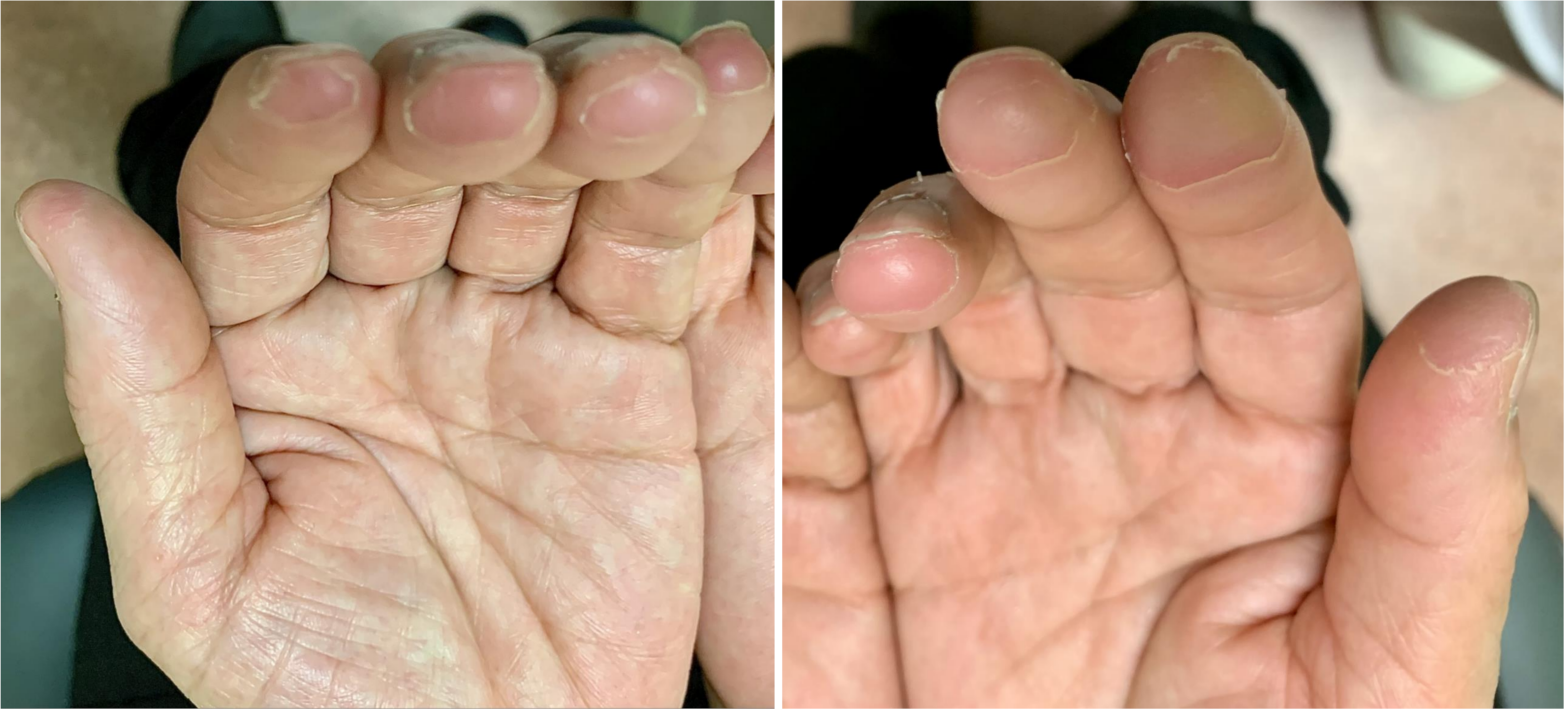
Epidermal exfoliation and keratotic changes are seen on both fingertips.

A 75‐year‐old woman presented with skin hardening and fingertip paresthesia. She had undergone left upper lobe lung adenocarcinoma cT2aN2M0 stage IIB resection 2 years before and had recurrence with right adrenal and right iliac metastases. A MET Exon 14 skipping mutation was identified and tepotinib 500 mg initiated 9 months ago. Physical examination revealed bilateral keratotic changes, fingertip paresthesia, and no foot lesions (Figure 1). Potassium hydroxide skin testing was negative. Tepotinib‐induced HFSR was diagnosed. The patient was treated with clobetasol propionate and improved after 70 days.

HFSR causes dysesthesia, erythema, and hyperkeratosis in areas of friction and pressure on the hands and feet.^1^ It is common in patients taking multikinase, BRAF, and EGFR tyrosine kinase inhibitors^1^; however, tepotinib‐induced HFSR has been reported in only other case with palmoplantar keratoderma‐like HFSR.^2^ The present case also showed hyperkeratosis of the fingertips, suggesting that hyperkeratotic lesions on the fingertips and toes may characterize tepotinib‐induced HFSR. However, further studies are required to confirm this finding.

Given that tepotinib may cause hand‐foot skin reactions, monitoring skin changes in the hands and toes during treatment is crucial.

## AUTHOR CONTRIBUTIONS

**Takafumi Yamano**: Writing of the original draft. **Toshihide Yokoyama**: Writing, reviewing, and editing. **Tadashi Ishida**: Supervision.

## CONFLICT OF INTEREST STATEMENT

None declared.

## ETHICS STATEMENT

The authors declare that appropriate written informed consent was obtained for the publication of this manuscript and accompanying images.

## Data Availability

Research data are not shared.
